# SELF-BLM: Prediction of drug-target interactions via self-training SVM

**DOI:** 10.1371/journal.pone.0171839

**Published:** 2017-02-13

**Authors:** Jongsoo Keum, Hojung Nam

**Affiliations:** School of Electrical Engineering and Computer Science, Gwangju Institute of Science and Technology, 123 Cheomdangwgi-ro, Buk-gu, Gwangju, Republic of Korea; Cincinnati Children’s Hospital Medical Center, UNITED STATES

## Abstract

Predicting drug-target interactions is important for the development of novel drugs and the repositioning of drugs. To predict such interactions, there are a number of methods based on drug and target protein similarity. Although these methods, such as the bipartite local model (BLM), show promise, they often categorize unknown interactions as negative interaction. Therefore, these methods are not ideal for finding potential drug-target interactions that have not yet been validated as positive interactions. Thus, here we propose a method that integrates machine learning techniques, such as self-training support vector machine (SVM) and BLM, to develop a self-training bipartite local model (SELF-BLM) that facilitates the identification of potential interactions. The method first categorizes unlabeled interactions and negative interactions among unknown interactions using a clustering method. Then, using the BLM method and self-training SVM, the unlabeled interactions are self-trained and final local classification models are constructed. When applied to four classes of proteins that include enzymes, G-protein coupled receptors (GPCRs), ion channels, and nuclear receptors, SELF-BLM showed the best performance for predicting not only known interactions but also potential interactions in three protein classes compare to other related studies. The implemented software and supporting data are available at https://github.com/GIST-CSBL/SELF-BLM.

## Introduction

In recent years, interest in identifying drug-target interactions has dramatically increased not only for drug development but also for understanding the mechanisms of action of various drugs. However, time and cost requirements associated with experimental verification of drug-target interactions cannot be disregarded. Many drug databases, such as DrugBank, KEGG BRITE, and SuperTarget, contain information about relatively few experimentally identified drug-target interactions [1–3]. Therefore, other approaches for identifying drug-target interactions are needed to reduce the time and cost of drug development. In this regard, *in silico* methods for predicting drug-target interactions can provide important information for drug development in a reasonable amount of time.

Various *in silico* screening methods have been developed to predict drug-target interactions. Among these methods, machine learning-based approaches such as bipartite local model (BLM) and MI-DRAGON which utilize support vector machine (SVM), random forest and artificial neural network (ANN) as part of their prediction model are widely used because of their sufficient performance and the ability to use large-scale drug-target data [4–9]. For these reasons, many machine learning based prediction tools and web-servers have been developed [10–13]. Especially, similarity-based machine learning methods which assume that similar drugs are likely to target similar proteins, have shown promising results [8, 9]. Although molecular docking methods also showed very good predictive performance, very few 3D structures of proteins are known, rendering docking methods unsuitable for large-scale screening [14, 15]. As such, a precise similarity-based method must be developed to predict interactions on a large-scale using the low-level features of compounds and proteins.

Previous similarity-based methods, such as the bipartite local model (BLM), Gaussian interaction profile (GIP), and kernelized Bayesian matrix factorization with twin kernel (KBMF2K), provide efficient ways to predict drug-target interactions and have shown very good performance [4, 16, 17]. BLM, which uses a supervised learning approach, has recently shown promising results using only similarities from each compound and each protein in the form of a kernel function. In the BLM method, the model for a protein of interest (POI) or compound of interest (COI) is learned from local information, which means that the model uses its own interactions of the COI or POI. This local-approach concept has been used in other methods, such as GIP, BLM-NII and others [17, 18].

Although such methods show very good performance, certain problems remain. Most previously developed methods categorize validated interactions between drugs and target proteins as positive, while unknown interactions are categorized as negative when constructing a predictive model. However, unknown interactions are not truly negative interactions, as they include potential interactions that have not yet been validated as positive interactions. To address this problem, Xia *et al*. developed a semi-supervised learning method (LapRLS) that regards known interactions as positive and unknown interactions as unlabeled data [19]. Chen *et al*. developed an algorithm using a network-based random walk with restart approach (RWRH) [20]. However, these methods demonstrate good performance in a limited set of conditions, where the drugs or targets use a drug-target network-based similarity score (NetLapRLS and NRWRH). Because these approaches are limited in predicting the interactions of novel compounds or proteins that do not have any known target or drug information (e.g., newly synthesized compounds or mutated protein sequences), other approaches are needed.

**Fig 1:**
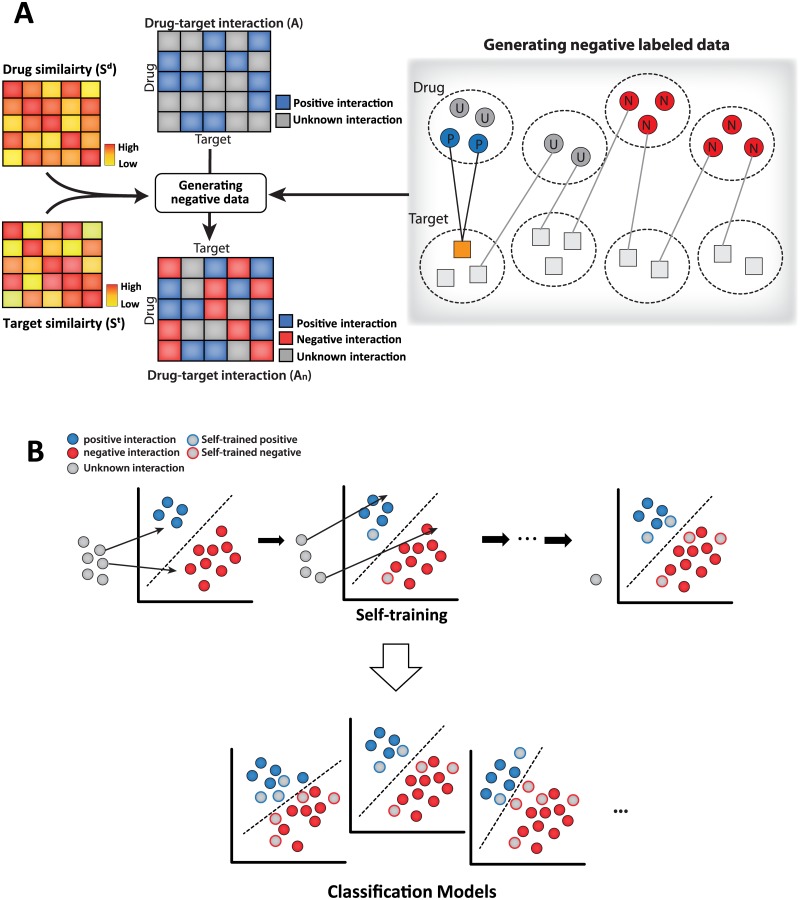
Overview of the proposed method. **(A)** From known information, drug-target interactions are classified into positive and unknown interactions (matrix A). Using similarity scores of drugs (matrix *S*^*d*^) and targets (matrix *S*^*t*^), we performed k-medoids clustering. If any of the drugs in a cluster do not interact with the cluster of the target protein, we considered the drugs in the cluster as having a negative interaction with the protein. Finally, drug-target interactions are classified into positive, negative and unknown interactions (matrix *A*_*n*_). Yellow rectangle: target protein, blue circle: drugs having positive interactions with the target protein, red circle: drugs having negative interactions with the target protein, gray circle: drugs having unknown interactions with the target protein. **(B)** A self-training SVM repeatedly trains the unlabeled data (unknown) as positive or negative. Finally, local classification models that can find potential interactions are constructed.

In this paper, we propose a drug-target interaction prediction method to predict potential interactions by using a modified BLM method. To classify unknown interactions into negative and unlabeled data, a clustering method was used before the training step [21]. Then, modified bipartite local models, termed self-training bipartite local models (SELF-BLMs), were constructed using a semi-supervised learning approach (self-training SVM) to improve a model’s ability to find potential interactions [22]. Fig 1 shows the overall process of the method. Finally, to train the model, we used a previous dataset for humans involving enzymes, G-protein coupled receptors (GPCRs), ion channels, and nuclear receptors from previous studies [23]. We then constructed another drug-target interaction data set that contained recently updated interaction information for performance validation. As a result, the number of drug-target interactions increased by approximately 60% for each type of protein. Our model showed good performance based on the area under the ROC curve (AUC) and the area under the precision-recall curve (AUPR) values of the updated dataset. In addition, our proposed method found the highest number of potential drug-target interactions compared to other related methods in most cases.

## Materials and Methods

### Drug-target interaction dataset for training

To train the model and cross-validate its performance, we used four types of drug-target datasets from humans, including enzymes, ion channels, GPCRs and nuclear receptors [23]. The data about the drugs, target proteins and drug-target interactions were derived from the KEGG BRITE, BRENDA, SuperTarget, and DrugBank databases [1–3, 24]. Table 1 shows details about the dataset information that was used.

**Table 1:** The number of drugs, target proteins, interactions and updated interactions of each type.

	Enzyme	Ion channels	GPCRs	Nuclear receptors
**No. of drugs**	445	210	223	54
**No. of target proteins**	664	204	95	26
**No. of drug-target interactions (previous)**	2,926	1,476	635	90
**No. of drug-target interactions (updated)**	4,449	2,029	1,268	168

### Drug-target interaction dataset for validation

Because the previous dataset was constructed in 2007, many newly identified drug-target interactions have since been discovered. To validate the performance power of predicting potential drug-target interactions, we updated newly identified interactions among drugs and target proteins that belonged to the previous dataset using the DrugBank, KEGG BRITE, and DsigDB databases [1, 2, 25]. The drug-target interactions obtained from DrugBank and KEGG BRITE databases were credible [1, 2], but the DsigDB database provided manually curated data and text mining data [25]. Because text mining data are massive and not credible, we selectively took manually curated data from the DsigDB database [25]. For this update, the numbers of updated interactions for each interaction type were 4,449, 2,029, 1,268, and 168, respectively. The number of drug-target interactions increased by approximately 60% for each type of protein. Using the updated dataset, we compared the performance and potential identification capability of each method. Table 1 shows a summary of the previous and updated dataset.

### Similarity metrics

The chemical similarities between drugs were calculated with the SIMCOMP method [26], which computes a global similarity score on the basis of common substructures between drugs using a graph alignment algorithm with the Eq (1)

$$S_{d}\left(d,d^{\prime }\right)=\frac{|d\cap d^{\prime }|}{|d\cup d^{\prime }|}$$ (1)

where d and d' are substructures of drugs

The structural information for the drugs was taken from the KEGG DRUG and KEGG COMPOUND sections of the KEGG LIGAND database [2].

The similarity between the proteins was calculated using a normalized version of the Smith-Waterman alignment score [27]. The normalized Smith-Waterman score between the proteins *P*_*A*_ and *P*_*B*_ was computed by the Eq (2)

$$S_{p}\left(P_{A},P_{B}\right)=\frac{SW(P_{A},P_{B})}{\sqrt{SW(P_{A},P_{A})}\times \sqrt{SW(P_{B},P_{B})}}$$ (2)

where SW is the Smith-Waterman alignment score

The amino acid sequences of the target proteins were derived from the KEGG GENES database [2].

### Generating negative interactions

To categorize unknown interactions as negative or unlabeled interactions, first, for each target protein, if a compound interacted with the target protein, we considered the interaction to be positive. We then clustered drugs and proteins by means of k-medoids clustering [21]. If any of the compounds in a cluster do not interact with the cluster of the target protein, we considered the compounds in the cluster as having a negative interaction with the proteins (Fig 1A). The remaining unknown interactions were considered to be unlabeled interactions, which are potentially positive interactions. These unlabeled interactions may later be classified as negative or positive interactions using the semi-supervised learning method (Fig 1B).

Because we used a k-medoids clustering method, an appropriate and consistent number of clusters was needed to train various datasets. In this study, we allowed to find one or two new positive interactions for each known positive interaction. Therefore, we set the number of unlabeled interactions to be no more than double the number of positive interactions. For example, if a protein has two known positive interactions, we set the maximum number of unlabeled interactions for the protein as four. The reason why we set the stringent limit for the number of unlabeled interactions is that too many unlabeled interactions could generate a decreased number of negative interactions, thereby resulting in a loss of negative data information for model construction. Therefore, we defined the number of clusters of drugs and targets as the resulting number when the overall number was divided by an integer, and we calculated the ratio of unlabeled interactions to positive interactions for the following integers N (one to ten). Table 2 shows that the ratio was between one and two when the number of clusters was the number of drug and target proteins divided by two for each protein type. Therefore, we finally set k to be the number of drugs and target proteins divided by two. The detail steps of generating negative interactions are described in Algorithm 1

**Table 2:** The number of drugs, target proteins, interactions and updated interactions of each type.

N	1	2	3	4	5	6	7	8	9	10
**Enzymes**	0	1.3	4.1	7.7	12.6	17.8	20.3	24	28.6	30.9
**Ion channels**	0	1.6	2.9	4	6.4	7.5	10.7	11.8	13.8	15.9
**GPCRs**	0	1.9	3.8	5.9	8	9.9	11.6	14.2	16.1	17.2
**Nuclear receptors**	0	1.5	3.7	5	7.6	9.4	9.6	11.9	11.8	12.7

### Bipartite local model

Bleakley *et al*. proposed a method called BLM to predict the interaction between a drug *i* and a target *j* [4]. BLM is described as follows. First, a local model for drug *i* is trained using an interaction profile of drug *i* and a similarity matrix of target proteins. Known interactions are regarded as positive, and unknown interactions are regarded as negative. Next, SVM constructs a classifier that distinguishes known interactions (positive) from unknown interactions (negative) using target similarity as a kernel. The model predicts the probability *p*_*d*_ (i,j) that a drug *i* and a query target *j* have an interaction by using the similarities between target *j* and the trained targets. Similarly, a local model for target *j* is trained using an interaction profile of target *j* and drug similarity. The model predicts the probability *p*_*t*_ (i,j) that a target *j* and a query drug *i* will have an interaction using the similarities between drug *i* and training drugs. Finally, we determine the predicted interaction value P(i,j) between drug *i* and target *j* with max(*p*_*d*_ (i,j), *p*_*t*_ (i,j)) or 0.5(*p*_*d*_ (i,j) + *p*_*t*_ (i,j)).

**Algorithm 1:** Generating negative interactions

1 **Generating negative interactions** (*A*, *S*^*d*^, *S*^*t*^);

  **Input :** Drug-target interaction matrix *A*,

     Drug similarity matrix *S*^*d*^,

     Target similarity matrix *S*^*t*^

  **Output:** Negative labeled drug-target interaction matrix *A*_*n*_

2 *k*_*d*_ ≔ |*D*|) / 2;   // D: set of drugs, *k*_*d*_: the number of drug cluster

3 *k*_*t*_ ≔ |*T*| / 2;     // T: set of targets, *k*_*t*_: the number of target cluster

4 *C*_*d*_ ≔ k-medodids(*k*_*d*_, *S*^*d*^);       //the set of drug clusters *C*_*d*_

5 *C*_*t*_ ≔ k-medodids(*k*_*t*_, *S*^*t*^);       //the set of target clusters *C*_*t*_

6 **for** *i* ← 1 **to** |*D*| **do**

7   **for** *j* ← 1 **to** |*T*| **do**

8    **if** *A*(*i*, *j*) = 1 **then**

9     *A*_*n*_(*i*, *j*) ≔ 1;                 //positive

10    **else**

11     *SD*_*d*__*i*_ ≔ set of drugs in the cluster containing *d*_*i*_;

12     *ST*_*t*__*j*_ ≔ set of targets in the cluster containing *t*_*j*_;

13     **if** *SC*_*t*_ *is not related* *SC*_*d*_ **then**

14      *A*_*n*_(*i*, *j*) ≔ -1;                //negative

15     **else**

16      *A*_*n*_(*i*, *j*) ≔ 0;               //unlabeled

17     **end**

18    **end**

19   **end**

20 **end**

21 **return** *A*_*n*_;

### Self-training support vector machine

To classify the unlabeled data, a self-training SVM was used [22]. In a local prediction step, the SVM model was constructed as a BLM using only labeled data. The unlabeled data were then classified by this model. If the unlabeled data passed the threshold, the unlabeled data were classified as positive or negative. The next step was to iterate this process until no unlabeled data failed to pass the threshold. Finally, the model used all labeled data as a local classification model to predict whether a compound targets proteins of interest and whether a protein is targeted by a compound of interest. The detail steps of constructing SELF-BLM models *M*_*t*_ for prediction of drug are described in Algorithm 2. In similar manner, SELF-BLM models *M*_*d*_ for prediction of target proteins are constructed.

**Algorithm 2:** SELF-BLM

1 **SELF-BLM** (*A*, *S*^*d*^, *S*^*t*^);

  **Input :** Drug-target interaction matrix *A*,

      Drug similarity matrix *S*^*d*^,

      Target similarity matrix *S*^*t*^

  **Output:** prediction model of target *M*_*t*_

2 *A*_*n*_ ≔ Generating negative interactions ((*A*, *S*^*d*^, *S*^*t*^));

3 *I*^*t*^(*i*) ≔ *A*_*n*_(:, *i*);            //A interaction vector of target *t*_*i*_

4 set $I_{L}^{t}\left(i\right)$;           //interaction vector of labeled data

5 set $I_{U}^{t}\left(i\right)$;          //interaction vector of unlabeled data

6 set $S_{L}^{d}$;            //Similarity matrix of labeled data

7 *M*_*t*_ ≔ $train(S_{L}^{d},I_{L}^{t}\left(i\right))$;         //Train a local model for *t*_*i*_

8 **do**

9   set ${S_{U}^{d}}_{XL}$;   //Similarity matrix of unlabeled data by labeled data

10   *P*_*U*_ ≔ $test(M_{t},{S_{U}^{d}}_{XL}$);     //Predict unlabeled interactions

11   **if** |*P*_*U*_| > *threshhold* **then**

12    change the unlabeled data to labeled data

13    set $I_{L}^{t}\left(i\right)$;

14    set $I_{U}^{t}\left(i\right)$;

15    set $S_{L}^{d}$;

16    *M*_*t*_ ≔ $train(S_{L}^{d},I_{L}^{t}\left(i\right))$

17   **end**

18 **while** *any unlabeled data is changed*;

19 **return** *M*_*t*_;

## Results

We trained the model using a previous dataset constructed by Yamanishi *et al* and validated the model using a previous dataset and an updated dataset [23]. Because some unknown interactions in the previous dataset turned out to be positive in the updated dataset, we can measure the potential identification capability of models by comparing the performance results.

First, we compared the performance of SELF-BLM with that of BLM [4], BLM-RBF, which includes drug-target network-based similarity using an RBF kernel, such as GIP or BLM-NII, and semi-supervised learning approaches, such as LapRLS, and NetLapRLS, which include network-based similarity [17–19]. For BLM and BLM-RBF, we used the modified source code that was originally given by the authors. We used the LIBSVM (v.3.21) to use SVM implementation [28]. When implementing SVM, the similarity matrices were used as a kernel without any modification. For parameters of SVM, values of C and gamma were assigned as 1 and 1 over number of features, respectively. For LapRLS and NetLapRLS, we implemented the methods based on the original paper. In the papers reporting these methods, BLM takes the maximum value between a drug-predicted value calculated using drug similarity and target predicted value calculated using target similarity, whereas LapRLS and NetLapRLS take an average value between the drug-predicted value and the target-predicted value; hence, we followed such approaches when we implemented these methods in the present study. SELF-BLM also takes the maximum value between the drug-predicted value and the target-predicted value.

Because the all compared models are local models, the models are repeatedly constructed using associated interactions for a given drug or protein. If the methods are evaluated in k-fold cross-validation, positive interactions are frequently not included in the training step. For example, in case of *epinephrine* drug, the drug has three positive interactions with 95 target proteins in the GPCRs dataset. Because of the small number of positive interactions, positive labels are often not included in the training set when the data is segmented into k-sets. Thus, we evaluated the performance of the models using leave-one-out cross-validation (LOOCV). However, in order to confirm robustness of our model, we also evaluated the performance using 10-fold cross-validation (S1 Table).

### Prediction performance

We calculated the performance of the interaction prediction in terms of the area under the ROC curve (AUC) value and the area under the precision-recall curve (AUPR) value. The AUC value is a common evaluation approach for binary classification problems. However, the large bias between the negative and positive training data sets often weakens the power of AUC values. Meanwhile, because it is important to classify the positive labels with high accuracy, the AUPR value may be a more appropriate indicator than the AUC value.

**Fig 2:**
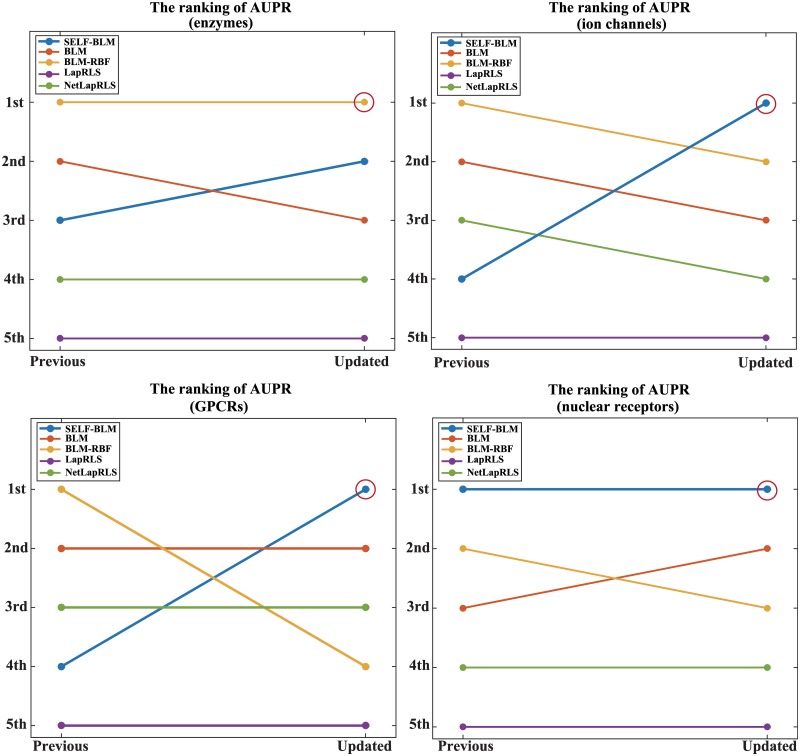
Rankings of AUPR trends by the different methods according to the updated dataset. In each panel, y-axis shows the rank representation of the AUPR value. A) the ranking in type of enzymes, B) the ranking in type of ion channels, C) the ranking in type of GPCRs, D) the ranking in type of nuclear receptor.

**Fig 3:**
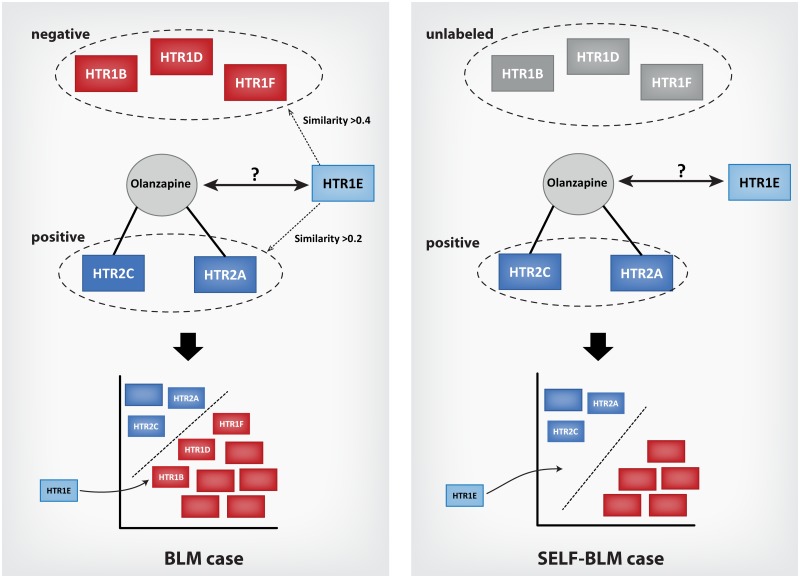
An example of SELF-BLM predicting the targets of a drug. In the previous dataset, it was known that proteins (HTR2A and HTR2C) bind to a drug (Olanzapine), but it was not known that other proteins (HTR1B, HTR1D, and HTR1F) also bind to the drug. Thus, in BLM, HTR2A and HTR2C are labeled as positive, and HTR1B, HTR1D and HTR1F are labeled as negative. Because the protein (HTR1E) is more similar to negatively labeled proteins than to positively labeled proteins, the protein is predicted to be negative. However, in SELF-BLM, these proteins (HTR1B, HTR1D, and HTR1F) are unlabeled. Therefore, the protein (HTR1E) is predicted as positive. There was no information suggesting that the protein (HTR1E) binds to the drug (Olanzapine) in the previous data, but it was later revealed that the protein indeed binds to the drug.

Table 3 shows the AUC and AUPR values of the five methods for the four type of proteins in each data set (previous and updated datasets). As the results show, the AUPR values of BLM-RBF were high in most cases when we used the previous dataset for validation. However, with the updated dataset, the AUC and AUPR values of SELF-BLM were the highest for most protein types, except for enzymes (Fig 2, S1 Fig). In Table 3, it is noticeable that the AUC and AUPR values tend to be decreased in the updated data. The main reason for this result is that some negatively labeled interactions changed into positive interactions when the dataset was updated. Therefore, there are previously predicted a fair number of interactions as negative, the AUC and AUPR values decreased in the updated data. For instance, to predict the interaction between target HTR1E and drug Olanzapine according to type of GPCR, HTR1E was considered similar to HTR2A (0.23) and HTR2C (0.23), which bind to Olanzapine (positive); however, HTR1E is more similar to HTR1B (0.43), HTR1D (0.44), and HTR1F (0.55), which do not bind to Olanzapine (negative) in the training dataset. Thus, BLM does not receive a high indication that HTR1E will bind to Olanzapine. On the other hand, with the SELF-BLM methods, these negative targets were regarded as potential targets, and some targets were considered unlabeled as a result. Thus, SELF-BLM yields high marks using unlabeled data generated by clustering and the self-training SVM method (Fig 3). Moreover, in the case of the previous dataset, because the interaction between HTR1E and Olanzapine is regarded as negative, SELF-BLM seems incorrectly predicting the interaction. This is the main reason why SELF-BLM shows decreased performance in some cases using the previous dataset. However, in the updated dataset, the interaction is now regarded as positive, and the performance of SELF-BLM thus increased.

**Table 3:** The AUC and AUPR values of the five methods for the four types of proteins in each validation set (previous and updated dataset).

		Enzymes		Ion channels		GPCRs		Nuclear receptors	
		Previous	Updated	Previous	Updated	Previous	Updated	Previous	Updated
**SELF-BLM**	AUC	**0.974**	0.859	**0.977**	**0.941**	**0.952**	0.914	0.890	**0.799**
**BLM**		0.968	0.846	0.972	0.923	0.94	0.893	0.869	0.767
**BLM-RBF**		0.974	0.880	0.975	0.903	0.930	0.880	**0.909**	0.792
**LapRLS**		0.954	**0.883**	0.960	0.915	0.894	0.872	0.816	0.778
**NetLapRLS**		0.960	0.869	0.958	0.928	0.926	**0.917**	0.867	0.789
**SELF-BLM**	AUPR	0.846	0.637	0.805	**0.762**	0.566	**0.614**	**0.625**	**0.573**
**BLM**		0.862	0.629	0.842	0.745	0.676	0.610	0.599	0.534
**BLM-RBF**		**0.891**	**0.652**	**0.922**	0.758	**0.709**	0.590	0.609	0.514
**LapRLS**		0.704	0.538	0.744	0.658	0.400	0.401	0.387	0.452
**NetLapRLS**		0.806	0.609	0.827	0.735	0.637	0.596	0.456	0.458

In addition, SELF-BLM could increase the prediction performance with the previous dataset by self-training unknown information. Because potential interactions are regarded as negative in the previous dataset, this approach makes it difficult for a model to be trained accurately. For example, in the case of predicting the positive interaction between target CHRM1 and drug Clozapine among GPCRs, the conditions are as follows. Target CHRM2 binds to Clozapine, and CHRM3, CHRM4, and CHRM5 do not bind to Clozapine (however, these targets actually do bind to Clozapine in the updated dataset). In similarity-based models, the CHRM1 model will choose a similar protein among targets. BLM does not indicate that CHRM1 will bind to Clozapine as CHRM1 is more similar to CHRM3 (0.45), CHRM4 (0.42), and CHRM5 (0.47) than to CHRM2 (0.42). In contrast, because SELF-BLM neither considers CHRM3, CHRM4, and CHRM5 as training data nor changes these targets to positive data beforehand, it predicts that CHRM1 will bind to Clozapine (S2 Fig). Therefore, SELF-BLM can yield high performance not only for the updated dataset but also for the previous validation dataset. Furthermore, additional experiment was conducted using up-to-dated drug-target information to show that the results are consistent in other dataset (see S1 File).

### Prediction performance for new interactions

**Fig 4:**
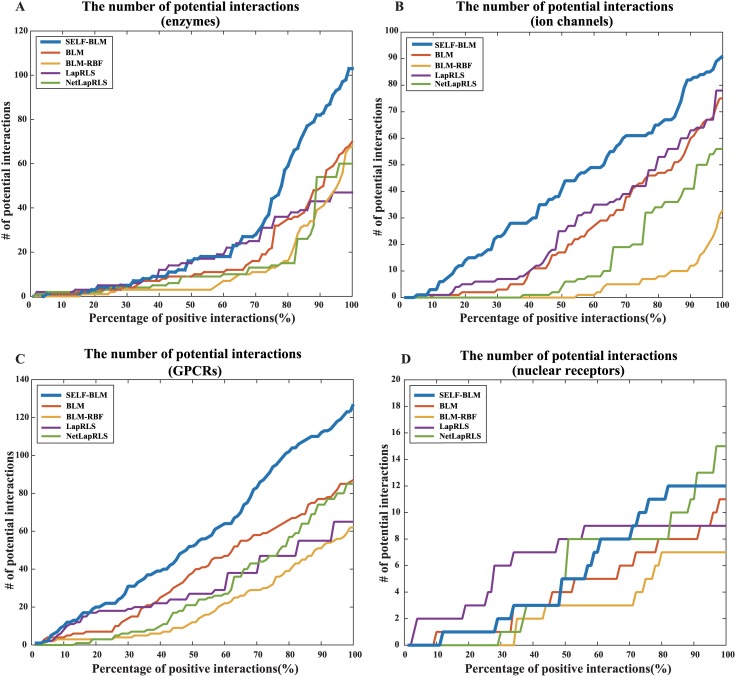
The number of potential interactions found by each method. X-axis represents the accumulated percentage of positively predicted interactions in each method, y-axis represents the number of correctly predicted potential interactions. A) The number of potential interactions according to type of enzyme. B) The number of potential interactions according to type of ion channel. C) The number of potential interactions according to type of GPCR. D) The number of potential interactions according to type of nuclear receptor.

Next, we evaluated the performance of models regarding potential interaction identification. We compared the number of potential interactions at each percentage of positive interactions from the top 1% to 100% of the ranked score. For example, the targets of GPCRs have 635 known interactions, so we set the positive as the top six (1%) to 635 (100%) from a total of 21,185 interactions, and the number of potential interactions were compared within the percentage range. As shown in Fig 4, SELF-BLM finds the most number of potential interactions than other methods for all of the protein types, except for nuclear receptors (see S2 File for details).

**Fig 5:**
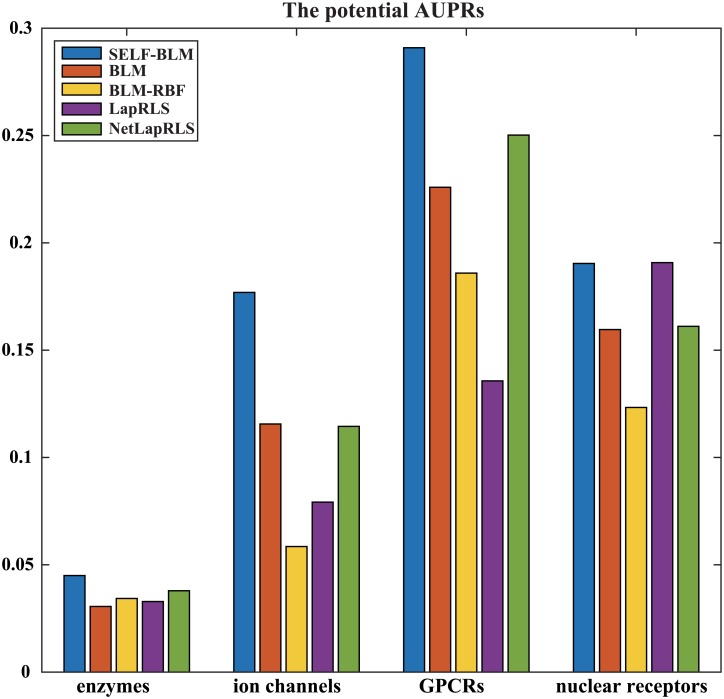
The potential AUPRs of the five methods for the four types of proteins.

Furthermore, we calculated the potential AUPRs of the four methods for the four types of proteins. In the potential precision-recall curve, positive labels were the potential interactions that were identified in the updated dataset, and negative labels were unknown interactions in the updated dataset. Therefore, we confirmed how the methods found the potential interactions simply by drawing a plot of the potential precision-recall curve (S3 Fig). The curves show that SELF-BLM finds many potential interactions with high accuracy. Thus, the AUPR of SELF-BLM was the greatest among the methods for all of the protein types, except for the nuclear receptor type. Fig 5 shows the potential AUPRs of the five methods for the four types of proteins.

In our results, BLM-RBF found few potential interactions and had low values of potential AUPR; also, the performance of BLM-RBF showed a greater drop than the other methods in most cases. Because BLM-RBF uses network-based similarities as an important factor for identifying a drug-target interaction, if a COI or POI had few interactions with the training set, the interaction similarities made it difficult to predict potential interactions of a COI or POI. This result shows that network-based similarity helps to find the interaction of a COI or POI that has a large amount of interaction information, but it is unsuitable for finding interactions of compounds or proteins for which little information about interactions is available. Although LapRLS and NetLapRLS are semi-supervised learning methods, we can confirm that these methods do not show good performance or a strong ability to identify potential interactions.

## Conclusion

In this study, we proposed a modified BLM, termed SELF-BLM, to accurately predict potential drug-target interactions. SELF-BLM uses k-medoids clustering and a self-training SVM algorithm to identify potential interactions among unknown interactions. To validate the performance of the method, we used benchmark datasets and updated recently verified interactions as potential interactions to the dataset using the DrugBank, KEGG, and DsigDB databases. Finally, we demonstrated the capability of SELF-BLM to predict potential interactions between drugs and target proteins. Notably, in most cases, SELF-BLM showed best validation performance with respect to AUC and AUPR for the updated dataset and found more potential interactions with high confidence prediction score compared to other methods.

In our study, we used a benchmark dataset for training to compare SELF-BLM with other methods and to validate its capability to identify interactions. However, as the research proceeded, various other similarity methods were developed. Like other similarity based-methods, SELF-BLM majorly depends on drug similarity and target similarity. Therefore, the performance of the model may be improved by using more-effective similarity methods such as kernel fusion method for various data fusion and/or efficient novel similarity features [29–31]. We emphasize that our SELF-BLM could show the best performance in the field of novel drugs or novel targets identification researches because our method does not require any known drug-target interaction information that is hardly known in novel molecules. Furthermore, in addition to drug-target protein interaction, it is important to deal with data imbalance problems or unlabeled data in many other areas so that, our method as well as the methods used in these areas can help to deal the problems [32, 33].

## Supporting information

S1 Fig Ranking of AUC trend according to the updated dataset among the methods. In each panel, y-axis shows the rank representation of the AUC value. A) the ranking in type of enzymes, B) the ranking in type of ion channels, C) the ranking in type of GPCRs, D) the ranking in type of nuclear receptors.
(EPS)

S2 Fig An example of SELF-BLM predicting the targets of a drug. In the previous dataset, it was known that a protein (CHRM2) bind to a drug (Clozapine), but it was not known that other proteins (CHRM3, CHRM4, and CHRM5) also bind to the drug. Thus, in BLM, CHRM2 is labeled as positive, and CHRM3, CHRM4, and CHRM5 are labeled as negative. Because the protein (CHRM1) is more similar to negatively labeled proteins than to positively labeled proteins, a predicted score of the protein is not high. However, in SELF-BLM, these proteins (CHRM3, CHRM4, and CHRM5) are unlabeled, therefore, the protein (CHRM1) is predicted as positive. In this process, SELF-BLM finds positive interactions confidently.
(EPS)

S3 Fig The potential precision-recall curve of the five methods for the four types of proteins. (EPS)

S1 Table The AUC and AUPR values of the five methods for the four types of proteins in each validation set (previous and updated dataset) using 10-fold cross-validation. (DOCX)

S1 File Additional experiments with up-to-dated drug-target interaction dataset. (PDF)

S2 File The number of potential interactions which are found by each method. (XLSX)
