# Supplementary material for: SELF-BLM: Prediction of drug-target interactions via self-training SVM
Source: PLoS One. 2017 Feb 13;12(2):e0171839. doi: 10.1371/journal.pone.0171839 (PMC5305209; doi:10.1371/journal.pone.0171839)
Supplement: S1 Table — (DOCX) [file pone.0171839.s004.docx]

S1 Table. The AUC and AUPR values of the five methods for the four types of proteins in each validation set (previous and updated dataset) using 10-fold-cross validation. The values represent the mean of 10-trials, and the values in parentheses represent the standard deviation of 10-trials.

|  |  | **Enzymes** | | **Ion Channels** | | **GPCRs** | | **Nuclear Receptors** | |
| --- | --- | --- | --- | --- | --- | --- | --- | --- | --- |
|  |  | Previous | Updated | Previous | Updated | Previous | Updated | Previous | Updated |
| **SELF-BLM** | AUC | 0.973  (0.001) | 0.860  (0.002) | 0.977  (0.002) | 0.939  (0.003) | 0.950  (0.002) | 0.911  (0.003) | 0.893  (0.006) | 0.799  (0.006) |
| **BLM** |  | 0.968  (0.001) | 0.846  (0.002) | 0.972  (0.001) | 0.922  (0.002) | 0.941  (0.003) | 0.888  (0.003) | 0.834  (0.014) | 0.750  (0.010) |
| **BLM-RBF** |  | 0.974  (0.001) | 0.879  (0.001) | 0.975  (0.001) | 0.901  (0.002) | 0.930  (0.003) | 0.877  (0.005) | 0.899  (0.014) | 0.780  (0.008) |
| **LapRLS** |  | 0.947  (0.001) | 0.878  (0.001) | 0.957  (0.001) | 0.911  (0.002) | 0.879  (0.003) | 0.863  (0.002) | 0.787  (0.017) | 0.759  (0.009) |
| **NetLapRLS** |  | 0.954  (0.001) | 0.863  (0.001) | 0.957  (0.001) | 0.927  (0.001) | 0.912  (0.001) | 0.908  (0.001) | 0.837  (0.003) | 0.773  (0.012) |
| **SELF-BLM** | AUPR | 0.840  (0.002) | 0.632  (0.001) | 0.799  (0.007) | 0.756  (0.002) | 0.557  (0.011) | 0.605  (0.002) | 0.600  (0.029) | 0.562  (0.004) |
| **BLM** |  | 0.851  (0.001) | 0.624  (0.001) | 0.833  (0.007) | 0.739  (0.002) | 0.646  (0.025) | 0.592  (0.004) | 0.550  (0.046) | 0.506  (0.008) |
| **BLM-RBF** |  | 0.890  (0.001) | 0.650  (0.000) | 0.920  (0.002) | 0.757  (0.001) | 0.705  (0.011) | 0.587  (0.002) | 0.586  (0.016) | 0.500  (0.003) |
| **LapRLS** |  | 0.699  (0.002) | 0.535  (0.000) | 0.739  (0.004) | 0.654  (0.001) | 0.390  (0.009) | 0.394  (0.002) | 0.367  (0.056) | 0.434  (0.011) |
| **NetLapRLS** |  | 0.801  (0.002) | 0.606  (0.001) | 0.825  (0.003) | 0.733  (0.001) | 0.622  (0.006) | 0.586  (0.001) | 0.440  (0.012) | 0.449  (0.003) |
