# Supplementary material for: SELF-BLM: Prediction of drug-target interactions via self-training SVM
Source: PLoS One. 2017 Feb 13;12(2):e0171839. doi: 10.1371/journal.pone.0171839 (PMC5305209; doi:10.1371/journal.pone.0171839)
Supplement: S1 File — (PDF) [file pone.0171839.s005.pdf]

Additional experiments with up-to-dated drug-target interaction dataset

Additional drug-target interaction dataset for training and validation

In this study, to prove the performance of our method using another dataset, the dataset of DrugBank database version 2, constructed in 2008, was used, and the dataset was updated using many up-to-date databases. This led to 971 drugs, 759 proteins, 2324 interactions, and 4016 updated interactions. Table A shows a summary of the previous and updated dataset. Because 698 of the interactions overlap with the interactions used in the study, we think the dataset is new and large. Therefore, the model was trained and tested with the dataset in a similar manner to the text.

Prediction performance

Table B shows the AUC and AUPR values of the five methods for the dataset. In SELF-BLM, the ratio of unlabeled interactions based on the number of positive interactions is approximately 1.3. The performance results provide evidence that SELF-BLM performs better with the updated dataset, and regardless of the datasets, SELF-BLM method is robust to find potential interactions compared to other methods.

Table A. The number of drugs, target proteins, interactions and updated interactions.

	**Total**
**No. of drugs**	971
**No. of target proteins**	759
**No. of drug-target interactions (previous)**	2324
**No. of drug-target interactions (updated)**	4016

Table B. The AUC and AUPR values of the five methods in the previous and updated datasets

	**AUC**		**AUPR**	
	**Previous**	**Updated**	**Previous**	**Updated**
**SELF-BLM**	0.911	0.888	0.432	0.442
**BLM**	0.895	0.868	0.490	0.433
**BLM-RBF**	0.882	0.880	0.441	0.413
**LapRLS**	0.880	0.875	0.215	0.230
**NetLapRLS**	0.889	0.881	0.318	0.293
